# Supplementary material for: Association between oral microbiome and seven types of cancers in East Asian population: a two-sample Mendelian randomization analysis
Source: Front Mol Biosci. 2023 Nov 21;10:1327893. doi: 10.3389/fmolb.2023.1327893 (PMC10702768; doi:10.3389/fmolb.2023.1327893)
Supplement: Supplementary file 1 [file DataSheet1.ZIP › Supplementary Materials/MR plots for tongue/tongue═╝/Colorectal cancer/pheno.3113_to_colorectal cancer_forest.pdf]

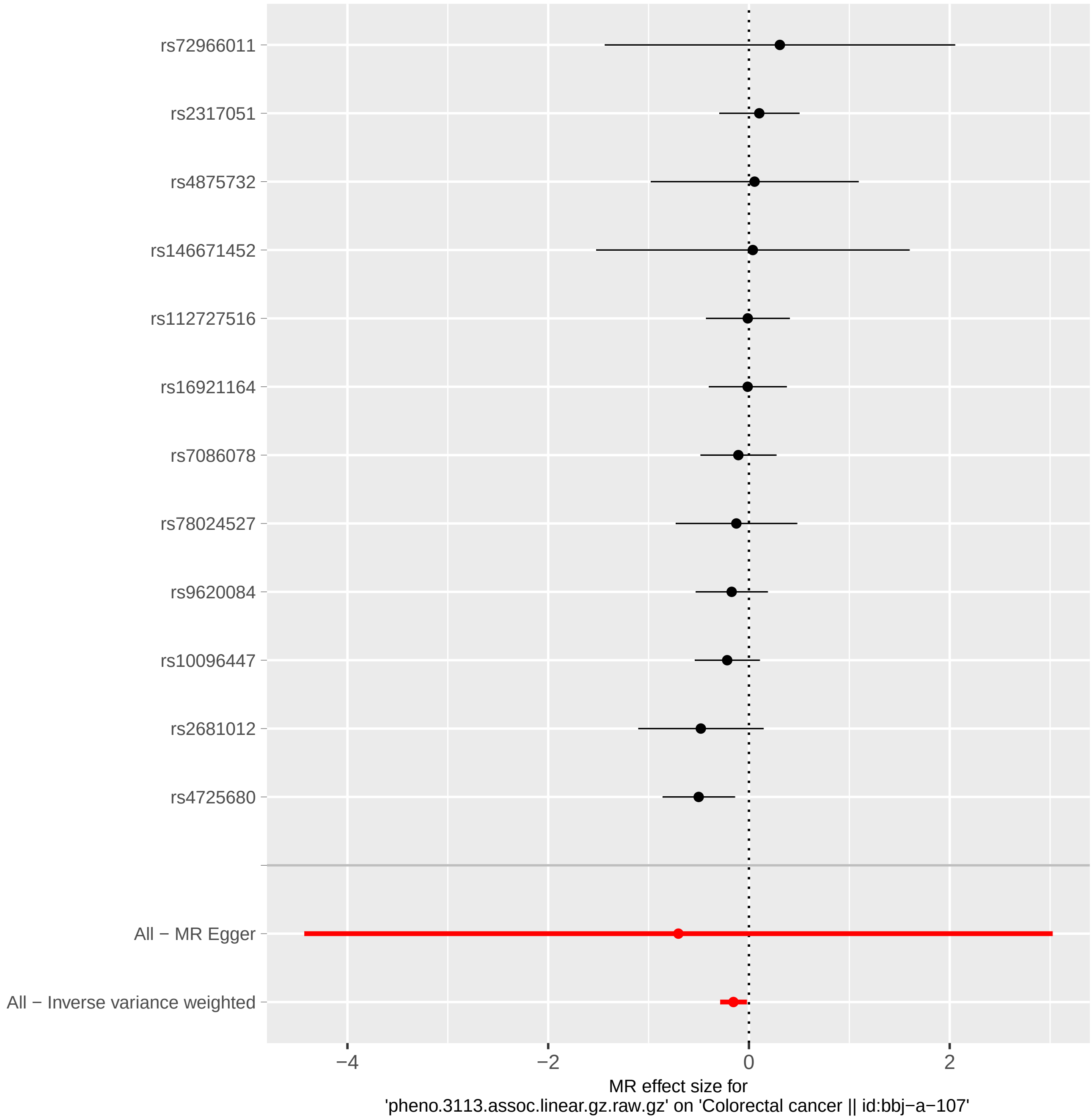
rs72966011
rs2317051
rs4875732
rs146671452
rs112727516
rs16921164
rs7086078
rs78024527
rs9620084
rs10096447
rs2681012
rs4725680
All − MR Egger
All − Inverse variance weighted
−4
−2
0
2
MR effect size for
'pheno.3113.assoc.linear.gz.raw.gz' on 'Colorectal cancer || id:bbj−a−107'
